# Network Neuroscience and the Adapted Mind: Rethinking the Role of Network Theories in Evolutionary Psychology

**DOI:** 10.3389/fpsyg.2020.545632

**Published:** 2020-09-25

**Authors:** Nassim Elimari, Gilles Lafargue

**Affiliations:** Department of Psychology, Université de Reims Champagne Ardenne, C2S EA 6291, Reims, France

**Keywords:** evolutionary psychology, modularity, structure–function relationship, hodotopy, network neuroscience, connectome

## Abstract

Evolutionary psychology is the comprehensive study of cognition and behavior in the light of evolutionary theory, a unifying paradigm integrating a huge diversity of findings across different levels of analysis. Since natural selection shaped the brain into a functionally organized system of interconnected neural structures rather than an aggregate of separate neural organs, the network-based account of anatomo-functional architecture is bound to yield the best mechanistic explanation for how the brain mediates the onset of evolved cognition and adaptive behaviors. While this view of a flexible and highly distributed organization of the brain is more than a century old, it was largely ignored up until recently. Technological advances are only now allowing this approach to find its rightful place in the scientific landscape. Historically, early network theories mostly relied on lesion studies and investigations on white matter circuitry, subject areas that still provide great empirical findings to this day. Thanks to new neuroimaging techniques, the traditional localizationist framework, in which any given cognitive process is thought to be carried out by its dedicated brain structure, is slowly being abandoned in favor of a network-based approach. We argue that there is a special place for network neuroscience in the upcoming quest for the biological basis of information-processing systems identified by evolutionary psychologists. By reviewing history of network theories, and by addressing several theoretical and methodological implications of this view for evolutionary psychologists, we describe the current state of knowledge about human neuroanatomy for those who wish to be mindful of both evolutionary and network neuroscience paradigms.

## Introduction

The emergence of functionally organized living systems is driven by causal processes that core assumptions and principles of evolutionary theory help to understand. Indeed, modern evolutionary biology allows the study of the gradual diversification and adaptation of said organized systems of various scales (e.g., cell clusters, organs, individuals) that occur through variation, selection, and retention of a considerable number of species-specific phenotypes. One of these principles is natural selection, a blind yet non-random selection process of genetic mutations, developmental patterns, and phenotypic plasticity mechanisms that provided survival or reproductive advantages in the form of adaptative responses to recurrent evolutionary problems. Given their beneficial effects on survival and reproduction, adaptive variations have a higher probability to be passed on to the next generation through various biological, behavioral, and environmental transmission pathways (Laland et al., 2015), which leads to the propagation of said adaptations as time goes on. As any other body organ, the brain is a functionally organized system and the physical embodiment of certain adaptations to recurrent evolutionary challenges. A large cluster of complex and interacting information-processing systems (varying between and across species) was selected over evolutionary time: they constitute what is commonly called the mind. Evolutionary psychology is the integrative study of these information-processing mechanisms—also called modules^1^ —and the adaptive behaviors they carry out in the light of insights drawn from evolutionary biology.

Understanding (1) why our mind happened to evolve in such a way, (2) what modules make us unique, and (3) what biological processes underlie these abilities are questions to which evolutionary psychology (as well as close fields such as evolutionary cognitive neuroscience) tries to offer a response. Interestingly, these questions mirror three of the main interests of neuroscience (and its various fields, e.g., cognitive neuroscience, neurogenetics, comparative neuroanatomy), the purpose of which is to study the neural architecture based on its phylogeny (i.e., how natural selection designed it), how this neural architecture presents itself in other living creatures, and what biological mechanisms govern its functional organization. Evolutionary psychology and neuroscience are two naturally converging fields that tackle similar issues with complementary levels of analysis. It is interesting to note that, from the first writings of pioneers in evolutionary psychology, a focus is made on mechanistic explanations for how neural circuitry produces cognition and behaviors. For instance, Cosmides and Tooby (1997) stated in their seminal paper that fully understanding any aspect of human cognition necessitates to answer four fundamental questions:

(1) Where in the brain are the relevant circuits and how, physically, do they work?
(2) What kind of information is being processed by these circuits?
(3) What information-processing programs do these circuits embody?
(4) What were these circuits designed to accomplish (in a hunter-gatherer context)?

Since then, many authors have emphasized the intrinsic informative value of brain architecture regarding the cognitive organization of a mind composed of evolved modules of various degrees of functional specialization (e.g., Duchaine et al., 2001; Bechtel, 2003; Bolhuis et al., 2011; Spunt and Adolphs, 2017). This focus on neural circuitry makes sense given the fact that the brain might be one of the most—if not the most—relevant vestige of phylogenetic and selective history of human behavior. It is common knowledge for evolutionary psychologists that the biological structure of living organisms varies along with the functions it subserves, a fact known since the time of Aristotle, the father of comparative anatomy, who studied the organizational principles of animal life by observing correlations between *form* and *function* (Blits, 1999; Catani, 2007). *Structure* and *function* (in the broadest sense of the terms) co-evolved influencing each other: variations in structure result in functional changes, and environmental pressures impose constraints on functional outcomes at the phenotypical level, which leads to a gradual modification in the anatomy of living organisms. The same applies for neuroanatomical structure. Thus, studying the brain may inform us on virtually any topic regarding evolved human cognition, such as (without being exhaustive) the processing features of modules, their distal causes, their universality, the way they shape human social environment, the environmental settings in which they evolved, or the other modules they co-evolved and interact with. In return, evolutionary models prove themselves to be indispensable to neuroscientists, who cannot dissociate the brain and its biological history, and should thus heavily rely on evolutionary insights to guide their theoretical and methodological frameworks. Since the structure–function question is as crucial to evolutionary psychologists as it is to neuroscientists, we believe sharing any advances made on this precise matter might be the key to bridge the gap between two complementary fields that should advance hand in hand toward a common comprehensive model of cognition. In this paper, we offer our contribution by briefly reviewing both history and the latest findings made on brain architecture in the field of neuroscience, as well as their methodological and theoretical implications on the path toward a greater understanding of evolved cognition. While this paper is broadly concerned with network theories, we will focus more closely on *hodotopy* (Catani and Ffytche, 2005), a theoretical account of brain–function relationships stemming from the field of neuroanatomy, that integrates both functional properties of segregated cortical territories (from the Greek *topos* = place) and connection pathways within the networks they may form (from the Greek *hodos* = path).

## Mapping Structure to Function in the Human Brain: History of Network Accounts

Understanding the neural substrates of cognitive functions has historically been a major challenge in cognitive neuroscience (Deacon, 1989; Catani and Ffytche, 2005; Catani, 2007). Numerous neuropsychological theories have made assumptions about brain organization through reflection upon the relationships between *structure* and *function*. Most of these theories can and have been classified in one of two major antagonistic paradigms: localizationism and holism. The former paradigm posits that functions are relatively independent processes, each of which is carried out by a given cortical territory. The latter highlights the importance of the brain as a whole over the role played by its parts. For more than two centuries, an ongoing and changing debate has (simplistically we might add) opposed these two paradigms for modeling an answer to the structure–function question. This debate can be traced back to the phrenological theory of Gall (1818) and the criticism brought by his archantagonist Flourens (1842) in the form of equipotentiality of the brain^2^. If the phrenologist view of Gall laid the foundation of localizationism, inheritance of this approach is best owed to Paul Broca’s work on language (Broca, 1861). It is thus without surprise that one of the most famous “*center of*” is Broca’s area, known by generations of students as “the language area.” Throughout the twentieth century, and until very recently, most studies have been carried out with a focus on the functionality of localized gray matter regions, disregarding brain connectivity and, among other things, the critical role played by white matter tracts (see for instance Fields, 2008; Wang and Olson, 2018; Wang et al., 2018 for discussions). Recent development in cognitive neuroscience has rendered obsolete this attempt to match each function to its segregated brain structure, which must be reconsidered in favor of a network-based approach: a function is the product of structurally and functionally interconnected brain areas. Given the emphasis made on connecting pathways in network models, it may seem at first glance that they tilt more toward holistic theories than toward localizationism. Nevertheless, hodotopy actually encompasses and reconciles both paradigms, as it focuses on both parcelation and functional properties of cortical territories, as well as on their relationships within the distributed networks they can form.

The divergence between network-based and localizationist accounts appears clearer when interpreting the emergence of symptoms following focal brain lesions. From a localizationist point of view, loss of function after neural insult is thought to be underlain by prejudice to a structure that was specifically and almost entirely operating the said function. While from a hodotopic perspective, any harm to the network (whether it concerns the cortical areas themselves or their cortico-cortical/subcortical connectivity) can jeopardize the onset of a function. Consequently, these two paradigms make distinct predictions concerning the evolution of post-lesional symptoms (discussed in the next section as well): localizationism leaves little to no room for functional resilience (except for peri-lesional area recycling), whereas network approaches can account for various mechanisms of neural plasticity (up to a certain extent), happening through dynamic rerouting of information stream in the brain. Unsurprisingly, it is based on lesion studies that early historical criticism of localizationism has been made. Marie (1906) had for example reported cases of non-fluent aphasia in patients without any lesion in Broca’s area and patients with damage to Broca’s area without any speech deficits. More than a century later, Thiebaut de Schotten et al. (2015) revisited three classic cases of neurology that have historically nourished the localizationist hypothesis: Louis Victor Leborgne (Broca, 1861), Phinéas Gage (Harlow, 1868), and Henri Gustave Molaison (Scoville and Milner, 1957). By superposing three-dimensional computer-generated reconstructions of these patients’ lesions to an atlas of white matter connections, the authors concluded that a disconnection-syndrome approach (i.e., impairment resulting from disruption of communication between brain regions) provided a better explanation for the presence of deficits than gray matter lesions. For instance, damages in Louis Leborgne’s brain was not limited to Broca’s area as initially reported, and various white matter tracts connecting multiple frontal, parietal, and temporal regions were severely impaired. The physical integrity of the left superior longitudinal fasciculus III (connecting Broca’s area to Geschwind territory), the left long segment of the arcuate fasciculus (connecting Broca’s area to Wernicke’s area), and of the frontal aslant fasciculus (connecting the pre-supplementary motor area to Broca’s area) were especially harmed (76.6, 59.5, and 55% of these tracts’ integrity, respectively). Louis Leborgne’s speech symptoms did not result from a single lesion in Broca’s area, but from extensive damages to tracts that induced major deficits in large functional networks.

These three cases are interesting to discuss given the fact that they are emblematic of the classic view of brain organization in fixed segregated areas. But data described in lesion studies that can only be explained by connectional models are not scarce. For instance, Philippi et al. (2009) report the case of a patient with next to pure white matter damage in the right inferior fronto-occipital fasciculus and inferior longitudinal fasciculus that presented specific emotion recognition impairments. Oishi et al. (2015) report cases of patients with intact gray matter structures interconnected by a damaged right uncinate fasciculus, with extent of tract injury being directly correlated with impaired emotional empathy. Drane et al. (2015) also describe how the use of a minimally invasive approach to epilepsy surgery (i.e., stereotactic laser amygdalohippocampotomy), which preserves white matter circuitry, is instrumental to spare cognitive functions normally impaired after surgery. More broadly, it is increasingly accepted, after decades of certainty that post-stroke deficits were caused by cortical insult, that structural and functional disruption of connectivity is critical in explaining cognitive or behavioral disorders, as disconnection syndromes may mediate the majority of post-stroke impairments (Silasi and Murphy, 2014; Corbetta et al., 2015; Sathian and Crosson, 2015). In a similar vein, Duffau (2012) extensively discusses cases of patients displaying “frontal symptoms”^3^ resulting from lesions of non-frontal areas (Teixidor et al., 2007; Krause et al., 2012; Sanai et al., 2012), as well as from parietal (Roux et al., 2003) or cerebellar (Budisavljevic and Ramnani, 2012) tumors. Duffau (2012) also addresses evidences from electrostimulation mapping studies (which include intraoperative functional mapping in awake patients) showing that transient lesions in non-frontal cortices or associative fiber tracts such as the superior longitudinal fasciculus (Moritz-Gasser and Duffau, 2009) can elicit frontal symptoms.

Neuroscience is quickly moving toward a network-based conception of the human brain: researchers are not solely focusing on neural topological elements anymore, but on their connections too. Since 2005, neuroscientists are trying to compile an accurate structural description of the brain network architecture, termed the “connectome” (Hagmann, 2005; Sporns et al., 2005). Converging with the hodotopic approach, the overarching purpose of the field of “connectomics” is to define the brain’s network architecture based on both topology and dynamics (whether it concerns brain-wide or local cortical connectivity), and thus revolves around two main organizational principles that need not to be isolated from one another: functional segregation and functional integration (Sporns et al., 2005; Hagmann et al., 2008; Sporns, 2013; Sporns and Betzel, 2016). In layman’s terms, functional segregation refers to the selective activity of certain neuron populations in any given neural process. Functional integration concerns the way computational outputs of segregated regions interact in a dynamic and distributed fashion to mediate the emergence of a complex cognitive or behavioral process. By investigating connectivity at the levels of neurons (i.e., microscale connectivity), neuronal populations (mesoscale connectivity), and brain regions (macroscale connectivity), this network map of the brain will permit production of new parcelation diagrams (e.g., Beckmann et al., 2009; Cloutman and Lambon Ralph, 2012) and “*will provide a unified, time-invariant, and readily available neuroinformatics resource that could be used in virtually all areas of experimental and theoretical neuroscience*” (Sporns et al., 2005).

## Neural Plasticity and Recovery of Function

Studying direct outcome of neural insult on cognitive functions is not the only way to explore the network organization of brain architecture. Neural plasticity, i.e., the brain’s ability to reorganize its structural and functional architecture in response to endogenous or exogenous environmental pressures, also provides useful information on cognition and the way it relies on network dynamics within the connectome. A specific form of neural plasticity, post-lesional plasticity is defined as the phenomenon of cerebral reorganization following brain damage. Post-lesional plasticity studies, especially in patients who underwent brain surgery, have historically proven themselves to be some of the most reliable sources of empirical evidence providing understanding of the brain anatomo-functional architecture. Longitudinal studies of functional compensation following lesions that occur early in an individual’s development are among the most interesting. For instance, Smith and Sugar (1975) reported the case of a 26-year-old man with above-average language and intellectual abilities, despite a left hemispherectomy undergone at age 5. The fact that these incredible instances of brain reorganization have been mainly observed in younger children led many scientists to conclude that post-lesional plasticity needed to occur in an immature brain to allow for function recovery. On the other hand, the major irreversible impairments induced by damage to even restricted areas have been considered as a proof that (1) potential for plasticity must be negligible in adult brain, and (2) neural circuitry is organized in highly specialized segregated areas (Desmurget et al., 2007; Herbet et al., 2017). In fact, localizationism has thrived for most of twentieth century on studies based on examination of limited functional resilience following acute lesions, such as strokes, but recent data show functional recovery following massive cerebral lesions even in adults. On this matter, models derived from studies of WHO grade II gliomas (i.e., diffuse low-grade gliomas) have received increasing attention over the past two decades. Diffuse low-grade gliomas are slowly growing brain tumors that preferentially infiltrate associative white matter fibers (Mandonnet et al., 2008). The slow but continuous migration enables progressive functional reorganization to such an extent that when surgical resection of even large brain areas is undergone, functional sequelae remains minimal (Duffau, 2005, 2006, 2014). In fact, while over 70% of patients continue to elicit mild to severe functional impairments up to 11 years after acute strokes, 93% of patients walk free of functional impairments within a year after diffuse low-grade glioma surgery (Duffau et al., 2003; Varona et al., 2004; Desmurget et al., 2007). Thanks to their pathophysiological features (i.e., slow growth, migration along fiber tracts) diffuse low-grade gliomas provide some of the most spectacular cases of functional resilience that neuroscience literature has to offer. For instance, a recent study report the case of patient “PR” (Lemaitre et al., 2018) who underwent bilateral prefrontal resection (200 cm^3^ of brain tissue resected, including Brodmann area 10 thought to be at the top of brain hierarchical organization) and should have shown severe deficits in executive functions and metacognitive abilities (Fleming et al., 2010; Yokoyama et al., 2010; Baird et al., 2013). And yet, contrary to any prediction permitted by the localizationist framework, PR keeps displaying normal executive functions under standard neuropsychological assessment and above-average metacognitive abilities (Lemaitre et al., 2018). If functions—such as those encompassed in the term metacognition—were localized, it would be impossible for patient PR to still hold metacognitive abilities, let alone above-average ones. Probably more than any other data in the neural plasticity literature, investigations on functional resilience following diffuse low-grade glioma resections demonstrate the special relevance of hodotopy. As we mentioned before, conclusions drawn from functional outcome after neural insult has historically guided theories on brain organization (Scoville and Milner, 1957; Broca, 1861; Harlow, 1868). The neural plasticity literature keeps providing useful empirical findings with deep theoretical implications (Lafargue and Duffau, 2008; Herbet et al., 2016, 2017) and will most likely continue to play an important part in the future of theories on neural organization.

## A Conceptual Pitfall: The Equipotential Brain

When exposed to data on post-lesional plasticity or to a critical view of localizationism, one may erroneously conclude that the brain (especially the cortex) is composed of interchangeable or disposable structures that have no special significance, and that brain functions (apart from low-level perceptual or motor functions) are non-specialized faculties derived from brain tissue as a whole: a thesis otherwise known as equipotentiality. In fact, it is in the form of theoretical by-products of the equipotential thesis (e.g., field theories) that localizationism has historically met challenge (Tizard, 1959), which is why we need to address this thesis as a theoretical pitfall, since equipotentiality is arguably the furthest conceptual leap one could make from localizationism, and an erroneous one.

Data on post-lesional plasticity may especially leave the impression that the brain is a highly adaptable and universal learning machine that can re-learn to perform certain tasks with an entirely distinct set of structures, which may seem inconsistent with the notion of evolutionary preparedness of cognition: “how could evolution have prepared us to respond to recurrent evolutionary challenges if the brain can switch between structures to assume the same function?” But post-lesional plasticity should not be treated differently than any other adaptation, as it is nothing more than myriads of mechanisms that collectively respond to the recurrent challenge of brain lesions^4^. It is in response to such hazards that the brain evolved to become more and more efficient at recovering from insult. Its ability to switch between different structures is thus not in contradiction with the concept of evolutionary preparedness. Moreover, even with millions of years of brain lesions promoting natural selection of individuals with high-potential for post-lesional plasticity, the mechanisms it encompasses occur under so much constraint that it cannot be accepted as an argument against evolutionary preparedness. In fact, post-lesional plasticity does not rely on arbitrary recruitment of any intact structure as initially suggested by Lashley (1929), but on (1) peri-lesional areas recycling, (2) reorganization or reponderation of a lesioned functional network, or (3) inclusion of new areas in the network, usually a contralesional homolog (Grady and Kapur, 1999; Bonnetblanc et al., 2006; Chen et al., 2010). While functional compensation can occur through recruitment of another network (e.g., increased activity in visual areas to guide gestures of patients with sensorimotor lesions; Seitz et al., 1999), recovery of the function itself depends on plasticity within the lesioned network. If the localizationist framework has a hard time accounting for instances of functional recovery following massive resection of eloquent areas, it is a fallacy to rely on the equipotential thesis or its corollary, a brain free of specialized structures where large random chunks of cortical tissue underlie a small number of ill-defined domain-general functions. Brain network organization is undeniably subject to stringent anatomical and connectional constraints that bias the onset of functions, as well as their recovery in cases of functional resilience. The hodotopic perspective remains a more suitable and balanced approach of cognition to account for the formidable potential of the brain to resume function after extensive neural lesions.

## An Integrative Network-Based Approach to Evolved Cognition: Methodological and Theoretical Implications for Evolutionary Psychology

Data presented here can only be properly understood within a hodotopic perspective, as the localizationist framework of functions cannot possibly explain occurrences of impairments resulting from pure damage to white matter fiber tracts (e.g., Philippi et al., 2009; Oishi et al., 2015), frontal syndromes following non-frontal lesions (Duffau, 2012), or preservation of executive functions and above-average metacognitive abilities following bilateral prefrontal resection (Lemaitre et al., 2018). Brain functions have to be conceived as dynamic processes arising from the integration of information within and between networks formed by interconnected clusters of cortical and subcortical neurons (Mesulam, 1990, 2005, 2008, 2009; Sporns et al., 2005; Badcock et al., 2019; Herbet and Duffau, 2020). New models of cognitive architecture derived from advances made in network neuroscience are now emerging, such as the hierarchically mechanistic mind (Badcock et al., 2019) or the meta-networking model (Herbet and Duffau, 2020). These models rest on the same assumption: the mind is a hierarchically organized system of nested, increasingly complex modules^5^, ranging from low-level highly specialized automatic mechanisms processed by relatively segregated and local networks to high-order flexible processes subserved by widely distributed networks. The repeated encapsulation of smaller modules in larger ones leads to a progressively distributed network organization mediating the onset of increasingly complex cognitive outcomes. Thus, the most complex, goal-directed, flexible, phylogenetically recent, and developmentally adaptable functions are thought to result from the global integration (between-network) of locally integrated information (within-network) derived from outputs of relatively specialized areas.

As we developed in the previous sections, this hodotopic perspective is not a recent one. Under different names, brain theories assuming that mental phenomena are supported by dynamic interactions within and between neural networks has been around in neuroscience for a century and a half, which contrasts with the 15-year-old interest for what is now called hodotopy or connectomics. Models of brain anatomo-functional architecture that could be considered “hodotopic” were even produced as early as 1905. Campbell (1905) contemporary and rival of Korbinian Brodmann, provided an integrative functional anatomical approach, which challenged Brodmann’s view in at least one respect: Campbell insisted on white matter connections between cortical areas to guide its account of functional anatomy. As for any other scientific theory, the place and importance of hodotopy in the literature was greatly determined by the technical means available at any given time to demonstrate its relevance. Interestingly, in the early 1990s, Francis Crick and Edward Jones published an editorial in *Nature* entitled “Backwardness in human neuroanatomy,” in which they called for an increased awareness about our lacking knowledge regarding the human brain. The authors deplored that this shortfall had gone mostly unnoticed by the scientific community and highlighted the pressing need for new methods to study human neuroanatomy:

“*Clearly what is needed for a modern human brain anatomy is the introduction of some radically new techniques, but unless there is a general awareness of the need for them they are not likely to arise. [*…*] We wish we had more concrete suggestions for new techniques. [*…*] If this article stimulates someone to devise suitable new methods to solve these problems, it will have served its purpose.*” Crick and Jones, 1993.

In the years that followed this appeal, new methods have indeed been developed and we are now able to explore communication within and between neurocognitive systems. These methods led to the rediscovery of the century-old network-based approach of structure–function relationships. In the next sections, we discuss some of these methods, as well as their implication for evolutionary psychologists who are now trying to identify the neural substrates of evolved modules. It is worth noting that it is beyond the scope of this review to come up with an exhaustive list of every method available to explore brain connectivity. Here, we focus on a few functional, effective, and structural connectivity analyses, and discuss their advantages and limitations.

### The Rise of Neuroimaging

In the early 1990s, functional magnetic resonance imaging (fMRI) flourished and was increasingly employed in a considerable amount of studies that investigated the neuroanatomy of cognitive processes. fMRI encompasses several techniques derived from the same principle: tracking the local blood-flow variations that results from the increased requirement of activated neurons for highly oxygenated blood. Changes in the magnetic properties of blood that depend on its degree of oxygenation can be monitored, which allows to infer about brain activity in target regions of interest. Understandably, after decades of localizationism, fMRI was celebrated as the ultimate technique to map each function to its dedicated segregated neural structure. While the statistical methods employed in fMRI studies rapidly moved from the early reductionist designs, most fMRI experiments remained largely concerned with the assessment of local brain area activations. Although this kind of experimental design allows for the detection of cerebral regions involved in a particular task, activation fMRI is unsuited to examine how brain regions interact (i.e., functional integration) and may only inform about one organizational principle of the brain, namely functional segregation.

Scientific progress depends greatly upon researchers’ ability to decide what kind of methods, data, and analyses are required to provide theoretical explanations for a given phenomenon. As time and progress go on, certain practices become deeply entrenched, especially when they were part of a larger framework of concepts, assumptions, rules, and methods that yielded powerful explanatory value. Obviously, neuroscience is no exception, and methodological inertia also tends to hamper the development and subsequent appropriation of new conceptual frameworks. The recent development of network neuroscience motivated more research for the neuroanatomical basis of functions in the connectome. But habits of thought and practice resulted in some instances in the incomplete (i.e., purely theoretical) appropriation of a network-based perspective that was thus restricted to the mere knowledge that networks exist somewhere in the brain. In such cases, the course-correcting value of a network-based approach has been somewhat limited to a search for task-dependent activations of cortical areas that may (or may not) match a list of structures known to be implicated in a network. In other words, while both functional segregation and integration has been grasped on a theoretical level, functional segregation remains the studied target of choice, given the employed experimental designs. The results derived from activation studies are obviously not intrinsically flawed, but any conclusion regarding brain connectivity drawn from these results are speculative by nature, as one cannot discriminate between independent parallel activations and actual network organization of a neural system. In this section, we briefly review some recently introduced methods that are best suited for *in vivo* exploration of functional interactions between brain regions, as well as the usefulness of each type of connectivity (i.e., functional, effective, structural) investigated by these methods.

### Functional and Effective Connectivity

Let us say we conduct an experiment on kin detection, the ability to assess genetic relatedness based on contextual (e.g., perinatal association with an individual’s mother), perceptive (e.g., facial features), or affective cues (e.g., feeling of familiarity), to guide mating, altruistic, and harming behaviors (Lieberman et al., 2007; Bressan and Kramer, 2015). In this experiment, participants are presented with photographs of their children in an experimental condition, contrasted with a control condition with photographs of familiar non-related children. After data analysis, we find an increased activation in a set of regions distributed across frontal, temporal, and limbic regions in the kin condition. These results bear multiple questions: are these activations coincidental or do these regions form a functional network that carries out kin detection? What is the functional role of each region within this potential network? What is the functional significance of other possibly downregulated areas? How does each region modulate the activity of another? Exploring these questions is not a luxury, but an essential part of a network-based approach. They need to be addressed to determine whether the—up until this point—hypothetical network is really mediating the specific cognitive output investigated.

The first question refers to functional connectivity, defined as the statistical interdependence between remote neurophysiological events (Friston, 2011). In simple terms, analyzing functional connectivity helps to identify which cortical areas communicate during a task. Basically, at least two plausible conclusions can be drawn from task-dependent activations of multiple brain regions: (1) the coactivity of these areas is interactive in nature, as they work together and form a network of kin detection, and (2) one or more regions were activated in an independent parallel manner and may not be directly involved in kin detection *per se*. If these regions interact to form a kin detection network, then their activity should be more strongly related during kin detection than during the control condition. Once again, activation fMRI can only assess functional segregation, so one must rely on task-dependent functional connectivity analyses (for instance psychophysiological interactions analysis; Friston et al., 1997) to determine whether these activations reflect local individual computations carried out by highly segregated areas, or if the computational outputs of each region are integrated in a distributed and coherent manner. These methods make it possible to infer functional connectivity between segregated areas by figuring out if their activation correlate (i.e., increase and decrease in synch) over time. For instance, psychophysiological interactions analyses allow one to explore the relationship (quantified in terms of strength of regression of activity in a region on another) between a seed region of interest and voxels across the brain (see O’Reilly et al., 2012, for a tutorial on psychophysiological interactions analysis). Functional connectivity analyses are but one of many efficient ways to explore dynamics in neural networks; however, they lack the power to fully characterize functional integration. More specifically, they are not fit to draw conclusions on the direction of information flow (i.e., causality) and are thus restricted to investigation on statistical interdependence. Other appropriate means are to be used to conclude on directionality of functional connections, the so-called *effective connectivity* (Friston, 1994), such as dynamic causal modeling (the gold-standard method for assessing effective connectivity; Friston et al., 2003), independent components analysis (Hyvarinen, 1999), or Granger causality (Granger, 1969).

Investigating functional and effective connectivity is not just about fancy experimental designs, it provides unique insights that may clarify (and sometimes even challenge) previous assumptions about dynamic interactions between brain areas identified with classical activation studies. For the sake of illustration, let us take an interest in the face-processing network, thought to be highly implicated in kin detection (Platek et al., 2005; Platek and Kemp, 2009). The dominant model of face-processing, and arguably one of the most influential neurocognitive models in social cognition, is the one proposed by Haxby et al. (2000). By identifying face-selective areas (i.e., brain regions that selectively respond to faces compared with other objects, e.g., the fusiform face area, a subregion of the fusiform gyrus, termed this way in recognition of its putative modular face-specific processing; Kanwisher et al., 1997), Haxby et al. (2000) provided a useful framework to think about the computational aspects of face-processing implicated in various socio-cognitive skills such as identity recognition, emotion processing, or attractiveness evaluation. According to this model, face-processing is carried out by a double neural system composed of a core network (i.e., comprising the occipital face and fusiform face areas, as well as the posterior part of the superior temporal sulcus) deciphering facial features and an extended network of limbic and prefrontal regions implicated in the subsequent matching of face-related information to perceptual, semantic, and affective information about self and others. While this model emphasized a distributed neural organization of face-processing from the very beginning (parting ways with the segregated view of a face-processing module localized in the fusiform face area; Kanwisher et al., 1997; Kanwisher and Yovel, 2006), communication within the face-processing network remained largely unknown until recently. Studies that were specifically aiming at disambiguating connectivity patterns in this network brought new insight into its neural organization and helped to address unanswered questions (e.g., What are the specific pathways underlying face-related sub-processes? What is the unique contribution of face-selective areas in the network? Is communication between face-selective areas content-dependent?). For instance, Fairhall and Ishai (2007) found specific connectivity patterns mediating the co-activations of face selective areas. More specifically, the occipital face area exerts direct and separate influences on the other two core network areas, namely the fusiform face area and the superior temporal sulcus, suggesting that the core network is organized in a hierarchical feed-forward architecture. In addition, while face perception elicits activation in most regions included in the core and extended networks, the author observed stimulus-dependent variations in coupling between the two: the fusiform face area, but not the superior temporal sulcus, was exerting a strong causal influence on limbic and frontal regions of the extended network when processing emotional and famous faces, respectively. Based on these results, the authors proposed the existence of two neural pathways within the face-processing network, with a notable ventral pathway dedicated to the processing of changeable aspects of face stimuli, relying on long-range connections between frontal, limbic, and occipital regions. Multiple studies helped to further understand the development and architectural properties of the human face-processing system (e.g., Cohen Kadosh et al., 2011; Nagy et al., 2012), indicating for instance that multiple parallel fronto-occipital pathways can be recruited for processing distinct facial affects (Dima et al., 2011). A growing number of studies also demonstrate the special relevance of network models for explaining face-processing deficits such as prosopagnosia (i.e., the inability to identify faces) that are better explained by abnormal inter-areal connectivity patterns (e.g., Avidan and Behrmann, 2014; Lohse et al., 2016; Rosenthal et al., 2017) rather than lack of cortical activation since many prosopagnosic patients can display relatively normal face-related activations (Hasson et al., 2003; Rossion et al., 2003; Avidan et al., 2005). Unsurprisingly, the advances brought by functional and effective connectivity studies play a significant part in prompting authors to revise the existing framework of face processing (e.g., Duchaine and Yovel, 2015).

The take-away conclusion here is that investigating network properties of a function’s neural substrates should rest on more than mere observations of cortical activations in areas that were previously associated with a particular network. This “checklist” approach can especially prove insufficient since it relies on the implicit assumption that brain regions are uni-functional and domain dedicated, while in practice a neural structure can be recruited (and even serve as a core component) in multiple networks. For the past decade, the number of papers investigating functional or effective connectivity has been growing steadily in all fields of neuroscience. This trend was predicted by Friston (2011), which noted that the yearly increase in papers concerned with connectivity exceeded the annual increase in publications on cortical activations for the first time in 2010. With reason, the author thought that this reflected a shift in emphasis toward connectivity *per se*. Thanks to the development of these new methodological approaches in the neuroimaging literature, functional integration is now gaining the place it deserves as a fundamental principle of brain organization. Without using the proper means to study the role played by functional integration in modules, the network-based approach of evolved cognition is at risk to remain incomplete.

### Structural Connectivity: On the Importance of White Matter

Studying functional or effective connectivity will be of chief importance in the upcoming quest to elucidate the biological basis of evolved modules. However, as these methods are derived from gray matter co-activation patterns analyses, they refer to a rather abstract form of connectivity. To make sense of these results and to paint a more comprehensive picture of brain connectivity, one must take an interest in how these statistical correlations are mediated by anatomical connections between linked cortical areas, i.e., structural connectivity. Cortical areas are not magically connected but wired through a vast array of white matter bundles, myelinated axons that occupy nearly half the human cerebral volume (Fields, 2008). They constitute the tangible, structural basis of the connectome promoting functional integration between brain regions. It is not without reason that we insisted on white matter in this review: this brain’s infrastructure for inter-region communication has a special significance in both neuroscience and evolutionary paradigms. From early theories of distributed neural organization (e.g., Dejerine, 1895; Marie, 1906 for findings on language, or Campbell, 1905 for an early network-based account of functional neuroanatomy), to more recent theoretical revision derived from reinvestigation of the most iconic cases in history of neuroscience (e.g., Van Horn et al., 2012; Thiebaut de Schotten et al., 2015), it is based on conclusions regarding white matter circuitry that most great empirical findings nudging scholars toward a hodotopic perspective have been made. Moreover, the present human brain is thought to result to a large extent from an evolutionary shift characterized by an increased investment of neural resources toward white matter circuitry (Semendeferi et al., 1994; Zhang and Sejnowski, 2000; Semendeferi et al., 2002; Schoenemann et al., 2005; Smaers et al., 2010, 2011; Ardesch et al., 2019).

The perceived importance of white matter in human brain evolution stems mainly from recent work in comparative neuroanatomy (i.e., the study of how human neuroanatomical architecture sets itself apart from that of its closest evolutionary relatives, such as chimpanzees^6^), more specifically from comparative studies of fiber tracts (e.g., De Schotten et al., 2012; Eichert et al., 2019; Sarubbo et al., 2019). Before that, frontal lobe expansion was thought to be what made the human neocortex special (Semendeferi et al., 2002; De Schotten et al., 2012; Sherwood and Smaers, 2013), a century-old theory primarily based on Brodmann’s (1912) work. Consequently (and to a certain extent, with reason), the frontal lobe has been considered a hallmark of human evolution ever since, thus being traditionally associated with the loftiest of functions (e.g., language, creativity, intelligence, self-awareness, executive cognition). However, the human frontal lobe is not just a uniformly scaled up version of the analogous neural structure found in primates. Frontal lobe expansion occurred along with specific anatomical changes in its overall organization, characterized by a differential in cortical areas’ relative size (Rakic, 1988; Semendeferi et al., 2002) and cellular developmental patterns (Rakic, 2009), as well as an increase in cortical folding (Zilles et al., 1988) and synaptic density and complexity (DeFelipe et al., 2002; Emes et al., 2008). But most and foremost, an increase in frontal white matter relative size (i.e., frontal hyperscaling; Smaers et al., 2010; Smaers et al., 2011) is what seems to really distinguish us from our closest taxonomical relatives (Semendeferi et al., 1994; Zhang and Sejnowski, 2000; Semendeferi et al., 2002; Ardesch et al., 2019), with prefrontal white matter showing the most significant difference between human and non-human primates, while no significant difference is observed in gray matter (Schoenemann et al., 2005). The recent history of our brain’s evolution^7^ is one of growing importance of connectivity patterns in its overall neurofunctional architecture. All of this points to the fact that white matter (and thus structural connectivity) should be investigated more, as advent of most phylogenetically recent functional adaptations have resulted in large part from structural changes in white matter circuitry, notably from an increase of associative fiber tracts range which gradually interconnected more and more cortical and subcortical clusters (putative functional groups) of neurons.

Once again, it is beyond the scope of this review to list every method available to explore structural connectivity *in vivo*. Here, we describe two methods focusing on white matter circuitry that have provided valuable data and unprecedented evidences regarding brain connectivity: diffusion-weighted MRI and direct electrical stimulation. Diffusion-weighted MRI is a recent technique that is used to assess white matter circuitry in a non-invasive way. It rests on the following principle: the structural properties of cerebral tissues impose constraints that hinder the normally random motion (i.e., Brownian motion) of water molecules; thus, it is possible to infer on the architectural features of white matter circuitry by probing molecular diffusion. For instance, the tubular structure of myelinated axons restricts molecular diffusion in certain directions, leading to an anisotropic diffusion pattern, which means that molecules start to diffuse in a preferred direction, specifically, along the axis of the fiber tracts. Diffusion imaging helps to track these changes in molecular movements on a per voxel basis, and multiple model-based methods provide voxel-based structural metrics (e.g., fractional anisotropy, axial diffusivity, radial diffusivity) that inform on both microstructural properties and trajectory of fiber tracts. The most prevalent model-based method is diffusion tensor modeling, which gave its name to diffusion tensor imaging (Basser et al., 1994), a technique that has been especially influential because it provided researchers with the ability to perform virtual dissections of white matter pathways, to generate three-dimensional representations of the connectome (i.e., diffusion-based tractography, Basser et al., 2000; Poupon et al., 2000; Shimony et al., 2004). Since diffusion tensor imaging is a structural technique, it does not inherently inform about the functional role of white matter connectivity in cognition or behavior. However, when used in combination with behavioral assessments, diffusion tensor imaging becomes a powerful method to test mechanistic hypotheses regarding the neural substrates of evolved modules. For instance, Brethel-Haurwitz et al. (2017) combined behavioral measures of altruism with diffusion tensor imaging and functional connectivity analyses to investigate the biological basis of care-based altruism, costly behaviors aimed at improving the well-being of vulnerable and distressed individuals (Marsh, 2016). Care-based altruism is thought to have evolved from parental care in mammals because it promoted group-level benefits that out-weighted individual costs. Thus, some authors have argued that care-based altruism was supported by neural systems that heavily overlap with the mammalian parental care system, underpinning neurocognitive mechanisms that enable detection of infantile features, process cues of emotional distress, and trigger protective rather than self-preservative behaviors (Lishner et al., 2008; Preston, 2013; Marsh, 2016; Brethel-Haurwitz et al., 2017). Supporting this hypothesis, Brethel-Haurwitz et al. (2017) found that highly altruistic individuals (who had donated a kidney to a stranger and scored highest on behavioral measures of altruism) exhibited greater fractional anisotropy in the white matter pathway connecting the amygdala and periaqueductal gray, limbic, and midbrain regions (respectively) that are known to be part of a network underlying parental care (Preston, 2013; Marsh, 2016; Fischer and O’Connell, 2018). While diffusion tensor imaging obviously comes with its set of limitations, such as the inability to fully describe fiber crossing, kissing, splaying, branching, or twisting at the voxel level (Basser et al., 2000; Wiegell et al., 2000; O’Donnell and Westin, 2011), this technique can open original methodologic avenues to test evolutionary hypotheses.

Contrary to the non-invasive nature of diffusion tensor imaging, direct electrical stimulation is a surgical technique that allows exploration of *in vivo* relationships between mental processes and well-defined cortical or subcortical neural sites with unmatched spatiotemporal resolution. To do so, surgeons create virtual lesions that transiently disturb a mental process that is assessed in real-time with behavioral tasks. Primarily used in the context of awake brain surgery to individually map a patient’s anatomo-functional architecture, direct electrical stimulation aids surgeons to maximize resection (usually for tumorological or epileptological reasons) while minimizing postoperative deficits, the main concern being sparing neural tissue that is essential to quality of life. Albeit invasive, direct electrical stimulation is widely regarded as a uniquely precise and reliable method to investigate brain organization, as the ability to disturb in real-time the activity within a system provides great insights into the role played by each of its components (De Schotten et al., 2005; Mandonnet et al., 2010; Desmurget et al., 2013; Duffau, 2015; Herbet and Duffau, 2020). This surgical technique has been used for 150 years to probe functional roles of cortical areas, first in animals (Fritsch and Hitzig, 1870), then in humans (Bartholow, 1874), and is now being employed to investigate the network organization of the brain by assessing causal links between cognitive functions and white matter tracts. By applying electrical current to a well-identified fiber tract, the surgeon can temporarily disconnect distant but axonally linked brain areas, which induces specific deficits that allows her/him to investigate the functional role of certain connection pathways (De Schotten et al., 2005; Duffau, 2015; Herbet and Duffau, 2020). For instance, identifying functional disturbances such as conduction aphasia or semantic paraphasia following electrical stimulation of the left arcuate fasciculus and the left inferior fronto-occipital fasciculus, respectively, greatly contributed to our knowledge of the language network organization (Duffau et al., 2002, 2005; De Witt Hamer et al., 2011). In another vein, Yordanova et al. (2017) found with the use of this technique that face-based mentalizing (i.e., inferring complex mental states on the basis of face stimuli) was supported by two neural pathways in the right hemisphere, subserved by the inferior fronto-occipital fasciculus (connecting three previously mentioned areas, namely, the occipital face area, fusiform gyrus, and superior temporal gyrus with frontal regions) and the superior longitudinal/arcuate fasciculus. These conclusions are consistent with previously evoked findings, such as the fact that pure white matter damage to the right inferior fronto-occipital fasciculus can elicit specific emotion recognition deficits (Philippi et al., 2009). Despite its invasiveness, the unique way in which direct electrical stimulation allows us to explore functional connectivity suggests that collaborative research between neurosurgeons and evolutionary psychologists has a strong potential to provide important and unprecedented knowledge regarding the biological basis of evolved modules.

Diffusion-weighted MRI and direct electrical stimulation have provided a wealth of empirical findings demonstrating the importance of white matter in multiple domains of cognition (e.g., Duffau et al., 2005; Parker et al., 2005; Aron et al., 2007; Han et al., 2009; Borchers et al., 2012; Duffau, 2012, 2015). Decoding connectivity patterns that stem from white matter circuitry (the brain’s physical infrastructure for inter-region communication) is an indispensable part of the construction of a network-based model of any function, and thus any evolved modules. Given the recent evolution of the human brain, there is no doubt that investigating structural connectivity is bound to yield essential insights, especially regarding the most recent and complex evolved modules. However, and as Friston (2011) develops: “*effective connectivity depends on structural connectivity, but structural connectivity per se is neither a sufficient nor a complete description of connectivity.*” Effective and structural connectivity are complementary approaches that, combined within a same research agenda, will help to provide a mechanistic explanation for how brain networks mediate evolved cognition.

## The Future of Network Neuroscience in Evolutionary Psychology

Most theoretical revolutions are preceded and depend, more or less directly, on methodological revolutions. It is thanks to technological advances and new analytical approaches that the nineteenth-century view of interconnected brain regions underlying cognition and behavior has been revived and is now being embraced in multiple fields of psychology. Given the close ties between evolutionary psychology and natural sciences, evolutionary psychologists are inherently informed about most theoretical developments in the various fields of biology, including neuroscience, and therefore adopted a network-based perspective early on (e.g., Aboitiz and Garcia, 1997; Panksepp and Panksepp, 2000). Recently, a myriad of exciting new theoretical or empirical papers are being published that take an interest in the neural correlates of functions defined within an evolutionary framework such as kin detection, cooperation, altruism (e.g., kin-based, reciprocity-based, care-based), competition, or attractiveness processing (Platek and Kemp, 2009; Marsh, 2016; Wlodarski and Dunbar, 2016; Reimers et al., 2017; Yamagishi et al., 2017; Heckendorf et al., 2019; Platek and Hendry, 2019; Kou et al., 2020). At the moment, little to no studies employ functional, effective, or structural connectivity analyses (see for instance Brethel-Haurwitz et al., 2017 for an exception), the standard practice being the tracking of cortical activations using classic fMRI paradigms. Obviously, the methods described in this review are very recent, and their appropriation is part of an ongoing and understandably slow process (Friston, 2011; Lang et al., 2012; Wang et al., 2018). With this paper, we aim to modestly contribute to this process, on the path toward a greater purpose shared by both evolutionary scientists and neuroscientists: the mapping of evolved neurocognitive architecture and its dynamism.

It might be relevant at this point to recall the fourth question from Cosmides and Tooby (1997): “What were these circuits designed to accomplish?”, and to consider the importance of hodotopy in human adaptive history with the following question: “What were neural networks designed to accomplish?” The study of human evolution has long sought to identify which environmental conditions have played a critical role in the emergence of key human adaptations. Several habitat-specific hypotheses highlight the selective pressure exerted by the physical environment of early humans on their cognitive abilities. For instance, one of the most influential theories of the twentieth century proposed that early human evolution (e.g., bipedality, tool-making, highly encephalized brain) was a response to the challenges of an open savannah:

“*For the production of man a different apprenticeship was needed to sharpen the wits and quicken the higher manifestations of intellect—a more open veldt country where competition was keener between swiftness and stealth, and where adroitness of thinking and movement played a preponderating role in the preservation of the species. [*…*] Southern Africa, by providing a vast open country with occasional wooded belts and a relative scarcity of water, together with a fierce and bitter mammalian competition, furnished a laboratory such as was essential to this penultimate phase of human evolution.*” Dart, 1925.

This idea, dubbed the “savannah hypothesis,” is but one of many environmental hypotheses of human evolution. For example, the woodland hypothesis provides a contradictory model in which early hominins evolved to adapt to arboreal activity in tree-dominated settings (Clarke and Tobias, 1995), while other hypotheses highlight the harshness of cold environments in higher latitudes (see Potts, 1998, for an extensive review). However, when one tries to understand the role played by early humans’ environment in their adaptive history, asking “what ecological conditions” might not be as relevant a question as investigating “how fast were they varying?” This is the core theoretical account of the variability selection hypothesis (Potts, 1996, 1998): rather than emphasizing the influence of a particular set of environmental settings (e.g., savannah, ice age), this hypothesis proposes that flexible cognition and sophisticated survival strategies have been necessitated by the demands of increasingly changing habitats. In all likelihood, environmental novelty—originating from the large disparities in ecological conditions during the Pliocene and Pleistocene eras—has played a prominent role in the emergence of high-level flexible cognition. But the physical environment has not been the only key driver of human brain evolution. Evolutionary scientists argued as early as the 1960s that high-level cognition might be (at least partially) a by-product of social intelligence (e.g., Holloway, 1967; Humphrey, 1976). Akin to other primates, humans are exceedingly skilled at negotiating social environments, and theories such as the social brain hypothesis or the Machiavellian intelligence hypothesis emphasize the role of social—rather than physical—challenges as a selective force driving the emergence of neural and behavioral complexity. The social brain hypothesis (Dunbar, 1998) proposes that high-level cognition evolved as a means to navigate the social world of human and non-human primates that is characterized by large groups and an unusual complexity compared with those of other animals (including other mammals). This complex social environment brought its lot of challenges such as the need to infer intentions, recognize emotions, deceit others, communicate, form coalitions, teach and learn from others, or adjust altruistic behaviors, all of which were so cognitively demanding that they exerted selective pressure on primates’ intelligence. The Machiavellian intelligence hypothesis also highlights the role of social complexity as a primary driving force of brain evolution, with a special interest taken in sophisticated strategies aimed at achieving higher social status in a context of intense social competition (Gavrilets and Vose, 2006). Empirically testing these hypotheses customarily involves investigation of correlations between social group size and brain overall volume or gray matter density in specific brain areas. Indeed, there exists a well-documented correlation between social group size and the neocortex volume across multiple primate species, including humans (Dunbar, 1998; Dunbar and Shultz, 2007). Multiple studies have also explored correlations between social group size and gray matter density in several cerebral structures, in particular the amygdala (Bickart et al., 2011; Kanai et al., 2012; Von Der Heide et al., 2014), the orbitofrontal cortex (Lewis et al., 2011; Von Der Heide et al., 2014), or substructures of the anterior temporal lobe (Kanai et al., 2012; Von Der Heide et al., 2014). On a side note, it is worth noting that the first studies investigating the relationships between social group size and brain connectivity have been published in the last decade (Bickart et al., 2012; Hampton et al., 2016). For instance, Hampton et al. (2016) have reported that individual differences in structural connectivity within a network formed by the aforementioned neural structures (i.e., amygdala, orbitofrontal cortex and anterior temporal lobe) predicted differences in real-world social network size.

The question as to which environmental challenges (physical or social) contributed the most in human brain evolution is still debated: some authors think that social selective pressure explains most of the phenomenon (e.g., Bailey and Geary, 2009), while others estimate that complex societies, however cognitively demanding, constituted a rather negligible selective force compared with habitat-specific challenges (González-Forero and Gardner, 2018). Nevertheless, it appears that recent human adaptive history has been shaped by the selective pressure exerted by complex societies and radical shifts in highly varying environmental settings. These social and physical environmental conditions are interesting to consider in the context of the hodotopic perspective since, as we developed earlier, most research in comparative neuroanatomy indicate that our brain architecture evolved during the same period to increasingly rely on widely distributed networks. Thus, it is likely that most cognitive adaptations to these environmental conditions rest on functional networks and should be investigated as products of neural connectivity. Anderson (2014, 2016) has for instance made a similar claim, suggesting that the diverse repertoire of behavioral adaptations humans developed over time is subserved by transiently connected neural subsystems allowing for flexible and sophisticated responses to environmental demands; and that the later an adaptation emerges, the more likely it is to rely on networks, given the greater number and diversity of neural structures available to support the adaptation. Consequently, it is indispensable to adopt a hodotopic (or network-based) approach when investigating the biological basis of these late adaptations to social challenges and environmental novelty such as the modules implicated in (for the most obvious) language, tool-making, meta-cognition, future-oriented cognition, abstract reasoning, and social cognition.

Further more specific questions will also have to be addressed down the road. For instance, subtle temporal changes in connectivity occurring over short (seconds) or large (years) periods of time—the so-called dynamical functional network connectivity (Sakoğlu et al., 2010)—are now considered as a central property of neural network functioning (Calhoun et al., 2014; Kopell et al., 2014; Herbet and Duffau, 2020). The fMRI community has recently become aware of the need to question the long-held assumption that functional connectivity patterns are stationary over time. Researchers are now moving beyond methodological approaches averaging entire datasets usually spanning from 5 to 30 min (Calhoun et al., 2014), and are thus focusing on much shorter or longer time frames. This perspective (referred to as “Chronnectome,” Calhoun et al., 2014; or “Dynome,” Kopell et al., 2014), which takes into account dynamic changes in coupling within the connectome, will be especially relevant to study some phylogenetically recent, general-purpose cognitive abilities such as executive functions or creativity. For instance, cognitive flexibility, defined as the readiness to switch between distinct cognitive processes to adjust appropriately to environmental changes, can hardly be regarded as a well-defined specialized function that would be carried out by a specific neural network. Rather, it should be considered as a “meta-function” of sort that naturally emerges from the intrinsic properties of dynamic, adaptable, and multifunction systems such as the brain, one of these properties being—in the case of cognitive flexibility—the promptness to recruit new networks in response to new and unexpected environmental demands. Thus, an increase in between-network dynamic interactions is a more appropriate candidate as neural correlate of cognitive flexibility than topological patterns of functional connectivity within any given network (e.g., Douw et al., 2016). New evidence suggests that other general-purpose cognitive abilities, such as creativity, are also more relevantly investigated as products of cross-network interplay rather than outputs of a distinct neural network (e.g., Beaty et al., 2016, 2018a,b; Li et al., 2017). Investigating subtle or long-term changes in network dynamics might be the key to understand the most phylogenetically recent, developmentally adjustable human mental adaptations driving behavioral innovation that are characterized by their relative flexibility and moment-to-moment adaptability (e.g., language, social bargaining, tool making, tool use).

## Conclusion

In the spirit of Wilson’s (1999) call for greater *consilience*, that is, the unification of human knowledge to form a common network of explanations and conceptual frameworks across all fields; a growing number of researchers consider that synthesizing research of all dsciplines and integrating scientific insights is the challenge of our generation. As the integrative study of cognition and behavior in the light of insights drawn from evolutionary biology, evolutionary psychology provides one of the most compelling and valuable theoretical synthesis in modern psychological science. Consequently, and since one might argue that any cognition is at least partly evolved cognition, evolutionary psychology will presumably play a decisive role in painting an exhaustive and interdisciplinary consistent picture of the human mind. Neuroscience ought to share and help potentiate this aspiration for greater *consilience*, as well as a common purpose with evolutionary psychology: to understand the design of cognition and to map human neurocognitive architecture. Network neuroscience will thus be instrumental in this endeavor, given its essential but so far under operationalized role in providing more meaningful mechanistic explanations for how mental phenomena arise from neural tissue. New insights have been gained on connectional neuroanatomy from lesion studies, neuroimaging or electrophysiological techniques, as well as computational models, which revived the century-old network-based approach of neural organization. We are now able to perform virtual dissections of white matter tracts, disconnect remote linked areas and assess the outcome in real-time, or conclude on the directionality of information flow between the sub-components of a network. These methodological developments open up new opportunities for evolutionary psychologists as well, and a burgeoning field is now emerging from the convergence of network neuroscience and evolutionary psychology. However, network neuroscience is not an easy field to navigate. Maintaining up-to-date knowledge and keeping abreast of new methodological or theoretical progress requires considerable investments. Understandably, and to the best of our knowledge, studies that actually employ connectivity analyses when exploring the biological basis of evolved cognition are still seldom at the moment. Nevertheless, such studies are bound to be an important part of the future of both evolutionary psychology and neuroscience, a future where modeling evolved cognition will rest upon evidence-based knowledge stemming from converging multimodal data.

## Author Contributions

NE and GL drafted and approved the final manuscript. Both the authors contributed to the article and approved the submitted version.

## Conflict of Interest

The authors declare that the research was conducted in the absence of any commercial or financial relationships that could be construed as a potential conflict of interest.

## Footnotes

1 In this paper, we will use the term “module” in the sense repeatedly defined in evolutionary psychology (Cosmides and Tooby, 1997; Barrett and Kurzban, 2006, 2012; Barrett, 2018), where it refers to any information-processing unit designed by natural selection to deal with evolutionary problems, whatever its degree of automaticity, domain-narrowness, innateness, or overall flexibility, in contradiction with the traditional Fodorian notion of modularity.

2 Equipotentiality is a two-centuries-old hypothesis about the structural substrates of brain functions, initially proposed by Flourens, and resurrected by Lashley (1929). This hypothesis essentially states that the cortex is a unit that serves brain functions and that no one part of it has a special significance. Thus, brain functions (Lashley was for instance concerned with maze learning) are non-specialized functions derived from brain tissue as a whole, and any part of the cortex can participate to the administration of any function.

3 Frontal symptoms are a collection of cognitive (e.g., language, attentional, or executive impairments), behavioral (e.g., disinhibition, perseveration), and other miscellaneous neurologic symptoms, usually subsumed under the umbrella term “frontal syndrome,” that have long been thought to be underlain by damages to the frontal lobes.

4 We do not deconstruct the various mechanisms of neural plasticity here, for it would need an entire paper on the matter. But many types of lesions (e.g., head traumas, viruses, toxins, senescence, etc.) for different classes of neurons have obviously created a need for the natural selection of many types of post-lesional plasticity. Post-lesional plasticity should not be mistaken as a single uniform mechanism responding to the vague challenge of “repairing the brain.”

5 In network neuroscience, a module is defined as a cluster of densely inter-connected neural elements that share a common, specialized function. It may refer to neurons at the micro-scale or brain regions at the macro-scale.

6 Chimpanzees and bonobos are especially good candidates for comparative studies because they are more closely related to humans on a genetical level than they are to gorillas or orangutans, so much that some authors would rather categorize these two species as “non-human hominids” (Varki and Gagneux, 2017).

7 Genetical studies suggest that chimpanzees and modern humans share a common ancestor that likely lived between 4 and 8 million years ago (Patterson et al., 2006; Langergraber et al., 2012), which helps to fathom the temporal scale of the rather relative phrasing “recent history.”
